# Supplementary material for: Thalamus enables active dendritic coupling of inputs arriving at different cortical layers
Source: Nat Commun. 2025 Sep 19;16:8327. doi: 10.1038/s41467-025-64152-0 (PMC12449479; doi:10.1038/s41467-025-64152-0)
Supplement: Supplementary file 1 — Supplementary Information [file 41467_2025_64152_MOESM1_ESM.pdf]

Supplementary Figures and Legends

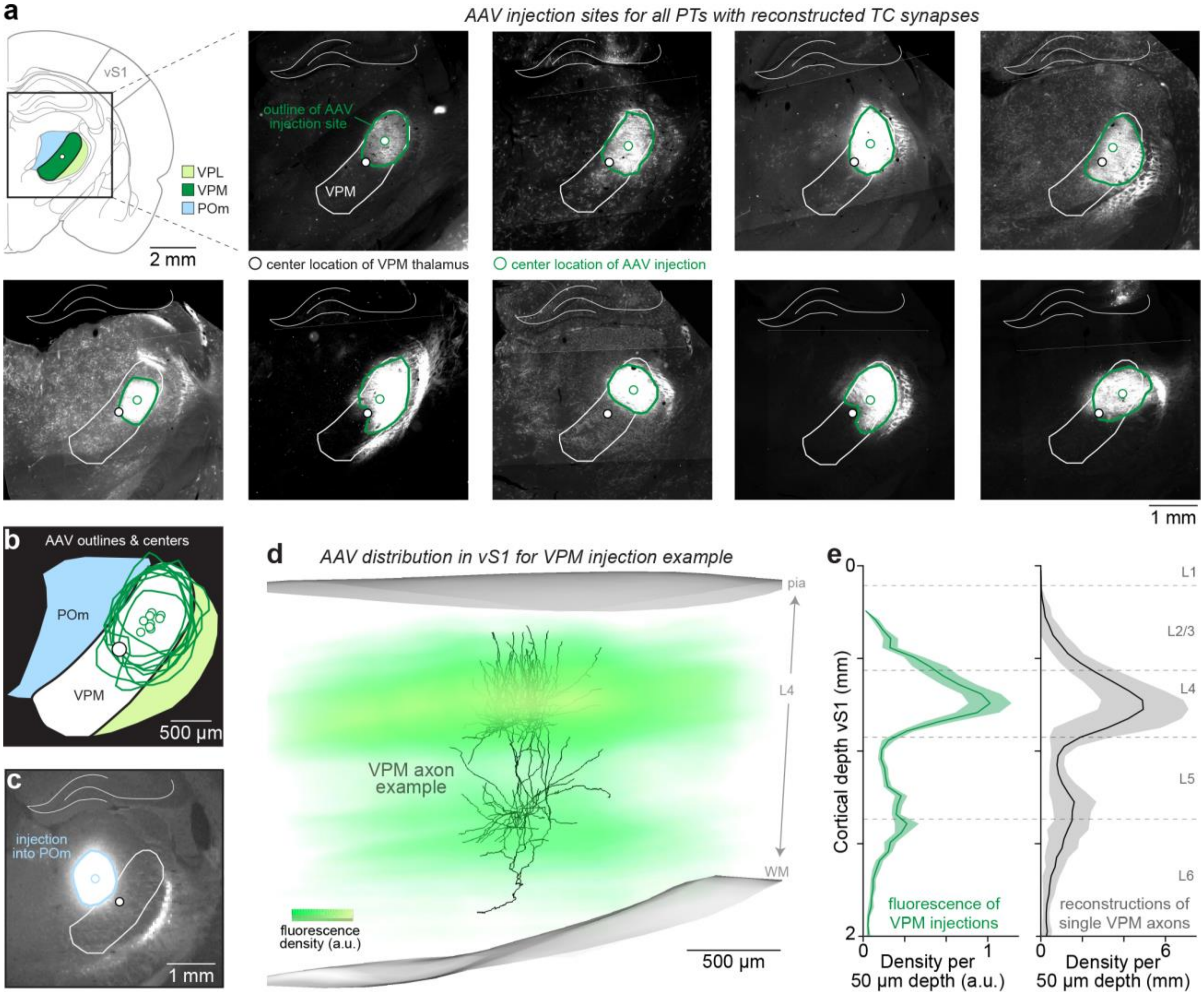

Fig. S1 (related to Fig. 1): Control data for AAV injections into VPM thalamus. a. For each rat (n=9) in which we identified TC synapses along the dendrites of *in vivo* recorded PTs (n=10), we took images of serial coronal sections that comprised the AAV injection sites and aligned them with the Paxinos Rat Brain Atlas[1] based on the outlines of thalamic nuclei, the hippocampus, cortex and brainstem. Image examples show that the AAV injection sites were located within to the VPM thalamus. Outlines were adapted and modified with permission from "The Rat Brain in Stereotaxic Coordinates, 6th Edition, George Paxinos and Charles Watson, Academic Press, Inc. USA (2006)." **b.** Overlay of all reconstructed injection volumes show that the center locations of the injections varied by less than ±58 µm across rats (N=9), and that the virus did not spread into the posterior medial nucleus (POm) of the higher-order thalamus. **c.** Example for injection that we targeted to POm, as reported previously[2]. **d.** Reconstruction of AAV fluorescence density across barrel cortex superimposed with axon reconstruction of a single relay cell in VPM thalamus[3]. **e.** Density profiles (mean ± STD) across the layers of the barrel cortex for AAV fluorescence (green; n=9 barrel columns) and single VPM axons (black; n=14 VPM axons; modified from[4]).

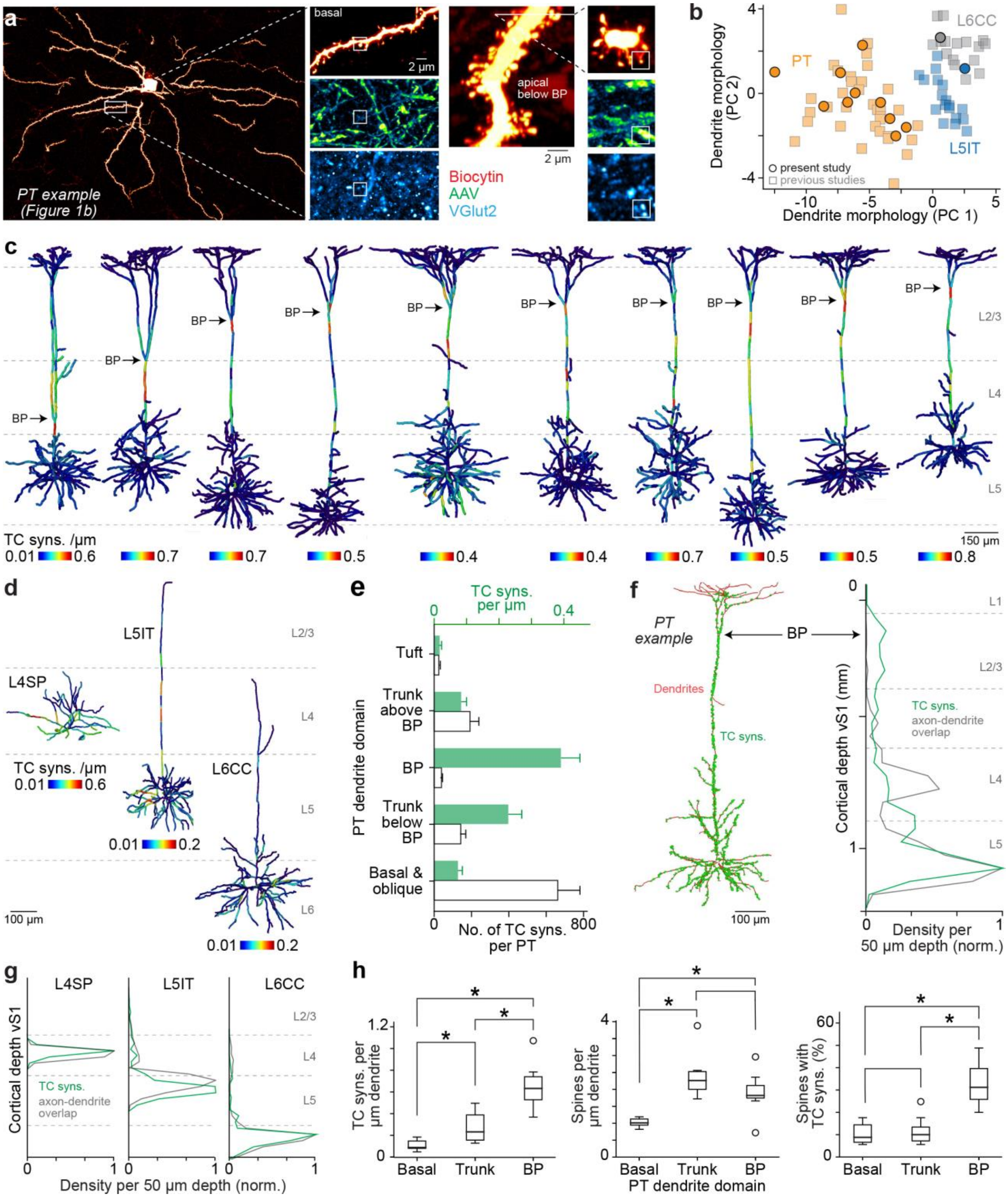

Fig. S2 (related to Fig. 1): Control data for AAV-based mapping of TC synapses along the dendrites of *in vivo* labeled PTs. a. Biocytin labeling of the PT example shown in **Fig. 1a-b**. Zoom-ins show super-resolution example images of spines along basal and apical dendrites (red), TC boutons along AAV axons (green), and VGlut2 puncta (cyan) **b.** Principal components (PCs)

of 21 dendritic features that discriminate between L5IT, L5PT and L6CC in rat barrel cortex[5]. Squares represent neurons that we reported previously[2, 5, 6], including PTs (n=22) with identified long-range targets in subcortical regions[2]. Circles represent the neurons reported in this study. **c.** Densities of TC synapses along the dendrites of all reconstructed PTs. **d.** Densities of TC synapses along the dendrites of one L4SP, L5IT and L6CC. **e.** Number (black) and density (green) of TC synapses along different dendritic domains (mean ± STD; n = 10 PTs). TC synapse distributions for each of the 10 PTs is shown on panel c. **f.** Left: 3D reconstruction of the dendrite (red) and TC synapses (green) of the PT for which the AAV injection site is shown in **Fig. 1a**. Right: Distributions of TC synapses along the dendrites of the PT on the left versus axo-dendritic overlap (normalized to the respective peaks) with reconstructions of individual *in vivo* recorded VPM cells[5]. Axo-dendritic overlap can account for the distribution of TC synapses along basal dendrites of PTs (i.e., in L5), but predicts higher than observed densities for apical oblique dendrites (i.e., in L4), and lower than observed densities for distal apical dendrites (i.e., in L2/3). **g.** Axo-dendritic overlap can account for the distributions of TC synapses along the dendrites of the L4SP, L5IT and L6CC in panel d. **h.** To demonstrate that the highest density of TC synapses around the BP of PTs does not reflect higher spine densities, we measured spine and TC synapse densities for three dendritic domains for all reconstructed PTs. Left: the density of TC synapses around the BP (0.81 ± 0.23 per µm of dendrite) is significantly higher compared to other parts along the apical trunk (0.34 ± 0.16) and to the basal dendrites (0.13 ± 0.05). The asterisks represent 1-way ANOVA tests with PostHoc Tukey multiple comparison correction for basal vs. trunk p=0.032, basal vs. BP p=0.0010, trunk vs. BP p=0.0010). Center: spine densities are not significantly different around the BP compared to other parts along the apical trunk, but significantly higher compared to the basal dendrites. The asterisks represent 1-way ANOVA tests with PostHoc Tukey multiple comparison correction for basal vs. trunk p=0.0010, basal vs. BP p=0.0012, trunk vs. BP p=0.086). Thus, while the fraction of spines that form TC synapses is largely constant across basal and apical dendrites (right panel), it is significantly higher around the BP. The asterisks represent 1-way ANOVA tests with PostHoc Tukey multiple comparison correction for basal vs. trunk p=0.90, basal vs. BP p=0.0010, trunk vs. BP p=0.0010).

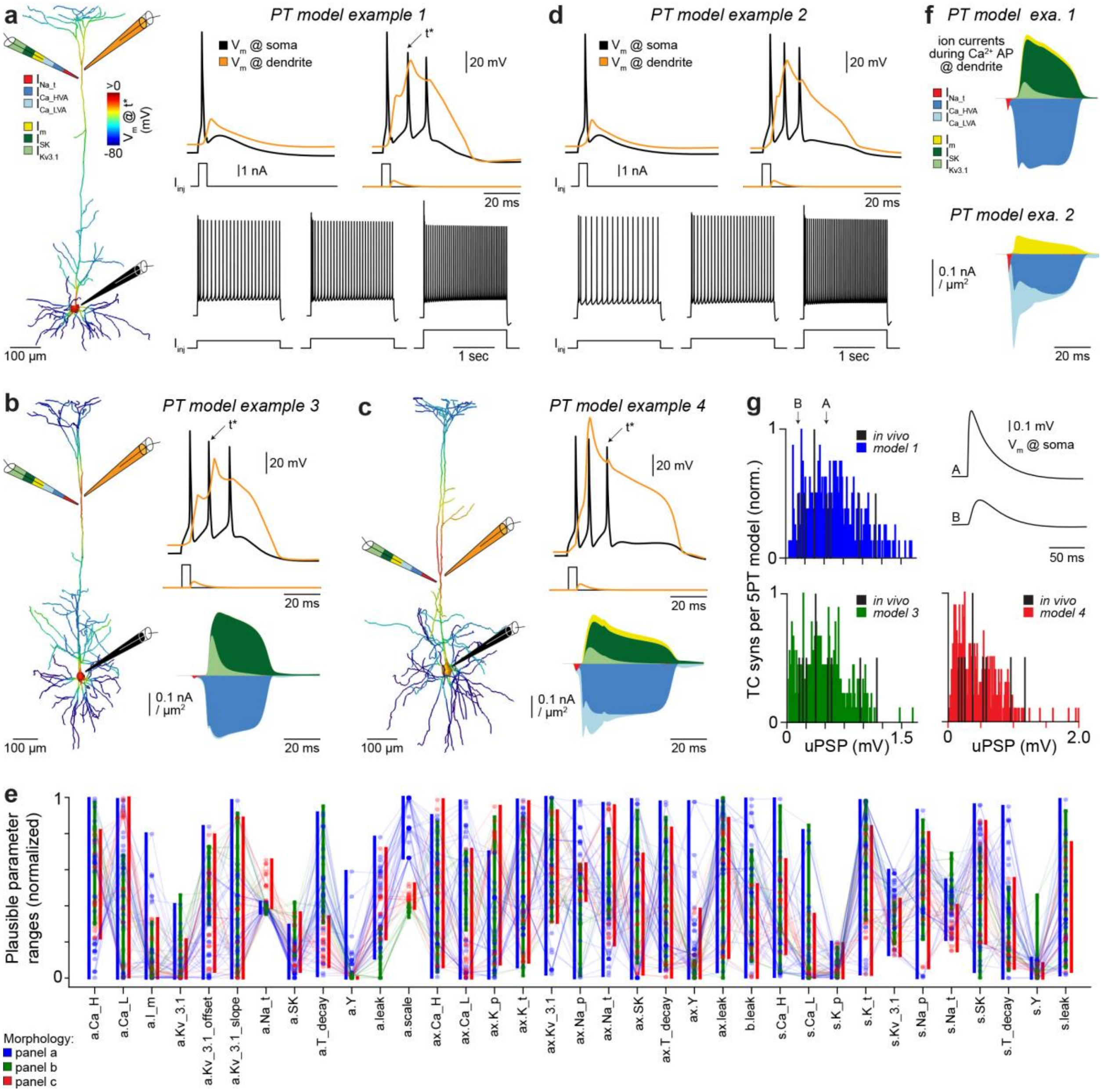

Fig. S3 (related to Fig. 3): Control data to account for parameter degeneracy in PT models. **a.** Example multi-compartmental model of the PT with the most superficial BP. For each multi-compartmental PT model, we simulated current injections into the soma (black) and/or calcium domain (orange) to capture the empirically observed dendritic (top) and perisomatic (bottom) physiology of PTs[7], including bAPs (top-left), Ca^{2+} APs and burst firing when inputs to the soma and Ca^{2+} domain coincide (top-right), and regular AP firing of increasing frequencies in response to sustained current injections of increasing amplitude (bottom). **b.** Example model of the PT with in-between BP (i.e., from the bulk of the distribution). **c.** Example model of the PT with the deepest BP. **d.** Second example model for the same PT as in panel a. **e.** Ranges of biophysical parameters across all models with acceptable dendritic and perisomatic physiology (n=68; most superficial BP: 40, in-between BP: 13, deepest BP: 15) representing the passive leak conductance and the

density of Hodgkin-Huxley type ion channels on the soma (s), basal dendrite (b), apical dendrite (a), and axon initial segment (ax). Parameters are normalized to their biophysically plausible ranges[7]. **f.** Both example models of the same PT morphology in panel a and d generate Ca^{2+} APs and bursts during coincidence detection equally well within the experimentally observed range (as do the example models with different PT morphologies in panels b and c), but utilize very different superpositions of ion channels to achieve these physiological responses. We plot hyperpolarizing currents at the BP during Ca^{2+} APs upwards: Ca^{2+}-dependent (SK), fast non-inactivating (Kv3.1) and muscarinic (M) potassium channels; depolarizing currents downwards: low- and high-voltage activated Ca^{2+} channels (Ca_LVA, Ca_HVA) and sodium (Na_t) channels. **g.** We simulated the activation of each reconstructed TC synapse on the dendrites of each PT model to optimize their respective peak conductance until their distributions matched the empirically observed uPSP amplitude distributions VPM and PTs[8]. Note: the strengths of synapses hence differ between models (n=68), but always meet the respective uPSP distributions observed empirically.

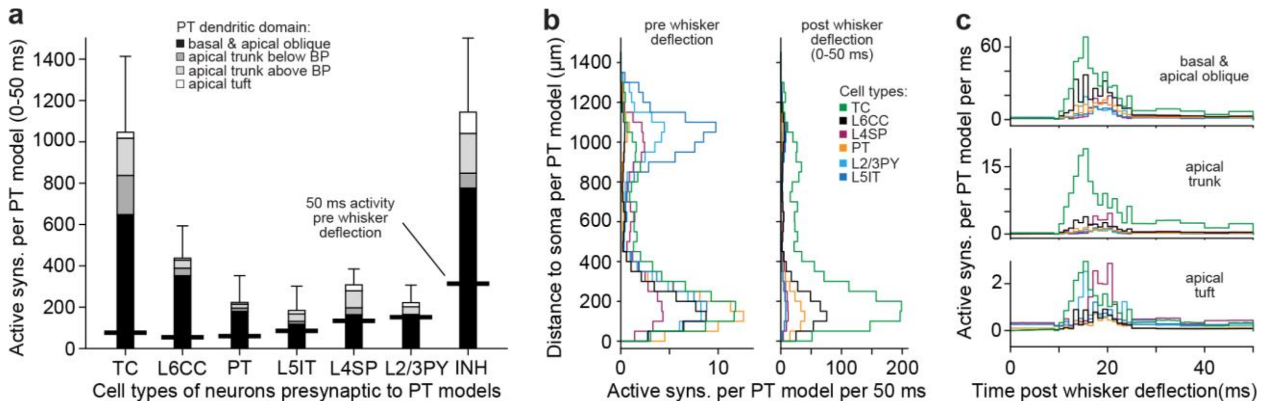

Fig. S4 (related to Fig. 3): Control data to account for variability of input to PT models. a. Quantification of spatiotemporal synaptic input patterns (mean ± STD) that impinge during pre- and post-stimulus periods of 50 ms onto the dendrites of 660,960 PT models embedded into network models of rat vS1 (one example for such a multi-scale model configuration is shown in **Fig. 3a/b**). The bold black lines denote the number of active synapses from each cell type during 50 ms preceding the stimulus. The gray shadings denote the respective numbers of active synapses during 0-50 ms post stimulus (i.e., passive whisker deflection by airpuff) onto different dendritic domains. Variability of spatiotemporal synaptic input patterns originates from the different dendrite morphologies of the PT models, their different embeddings into the network model, and the activation of their presynaptic neurons in the network model by generating Poisson spike trains based on our *in vivo* recorded firing rates for each cell type. **b.** Quantification of the same spatiotemporal synaptic input patterns as in panel a (mean), now resolved by dendritic locations of input. Note: despite generally low prestimulus firing rates during anesthesia, synapses from neurons across all layers and of all cell types imping onto PTs, with L5IT providing the majority of ongoing inputs to the apical tufts in the model during these experimental conditions (left). Furthermore, even though TC synapses from VPM represent generally less than 5% of the total number of inputs that PTs receive, they represent the majority of active synapses after stimulus onset during these experimental conditions (right). **c.** Quantification of the same spatiotemporal synaptic input patterns as in panels b (mean), now resolved by 1 ms time bins of input. Note: TC synapses dominate the sensory-evoked input to the trunk of PTs, whereas inputs to the proximal (i.e., basal and apical oblique) and distal (i.e., tuft) dendrites arise equally abundant from the EXC cortical types.

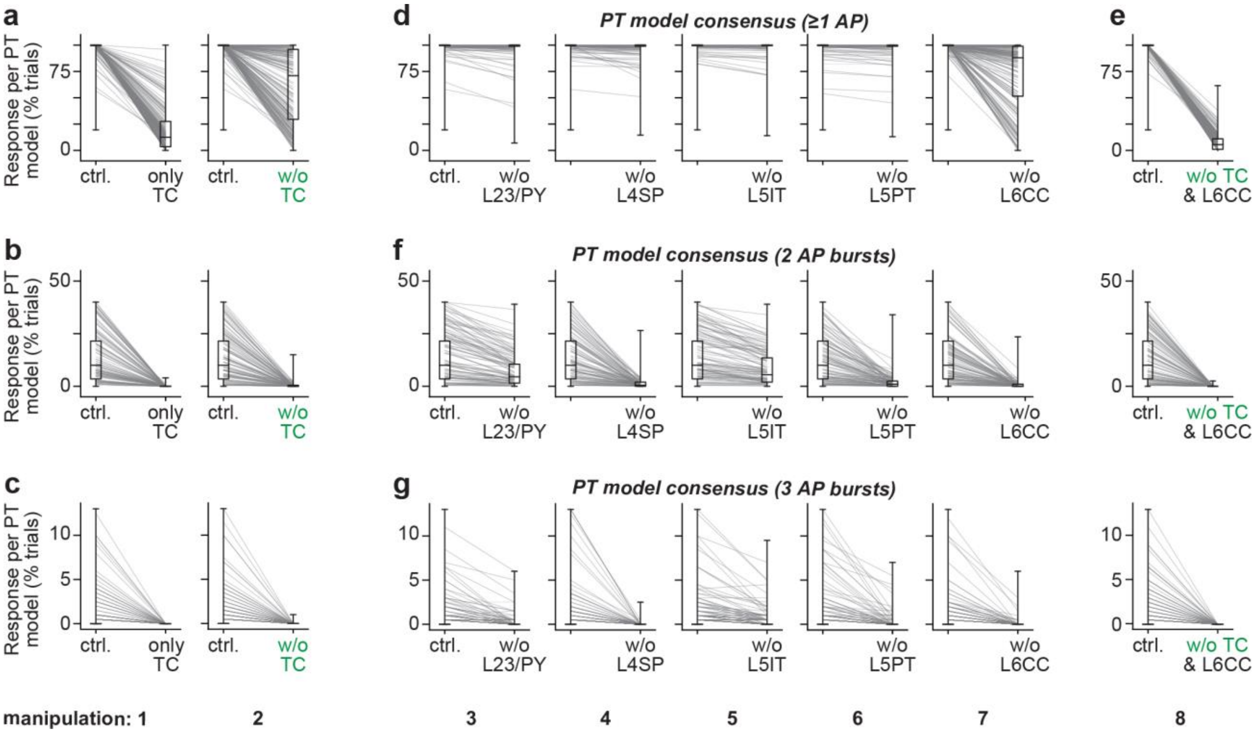

Fig. S5 (related to Fig. 4): Control data for *in silico* manipulations. a-c. Model consensus from all 20359 model configurations which exhibit burst responses: we deprived PTs from indirect sensory input via TC-driven excitatory neurons in the barrel cortex (manipulation 1) or from direct sensory input via TC synapses along their dendrites (manipulation 2). **d-g.** Model consensus from all 20359 model configurations which exhibit burst responses: we deprived PTs from indirect sensory input by each excitatory type in the barrel cortex separately (manipulation 3-7). **e.** Model consensus from all 20359 model configurations which exhibit burst responses: we deprived PTs from L6CC input and at the same time from direct sensory input (manipulation 8). In all panels, box plots represent medians and 25th to 75th percentiles. Whiskers extend to the full data range.

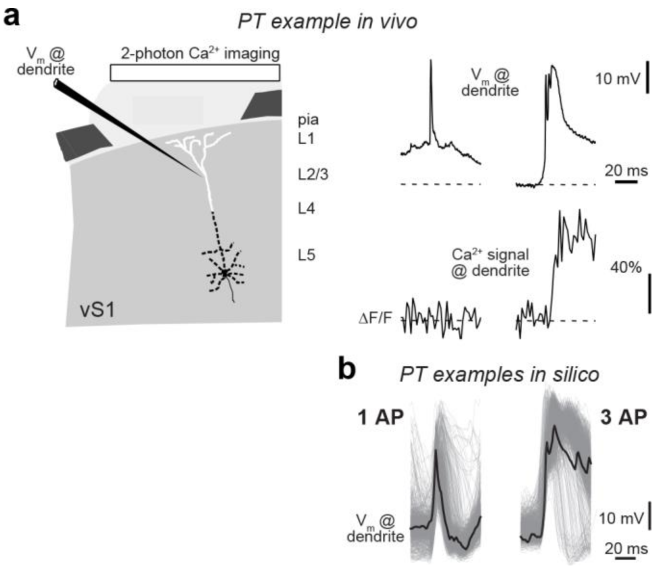

Fig. S6 (related to Fig. 5): *In silico* dendritic responses vs. *in vivo* dendritic Ca^{2+} signals. a. These panels were adapted from "Helmchen F, Svoboda K, Denk W, Tank DW. In vivo dendritic calcium dynamics in deep-layer cortical pyramidal neurons. Nat Neurosci 2, page 992, 1999, Springer Nature". Reproduced with permission from Springer Nature. This study showed whole-cell recordings and Ca^{2+} imaging of whisker-evoked responses near the primary BPs of PTs in the barrel cortex (vS1) of anesthetized rats[9]. These *in vivo* experiments indicated that sensory-evoked Ca^{2+} APs (i.e., *'complex'* potentials[9, 10]) result in detectable Ca^{2+} signals (right), whereas sensory-evoked *'fast'* potentials may not (left). **b.** Membrane potential *in silico* at the Ca^{2+} domain for 1,000 example simulations with sensory-evoked 1 AP responses (left) or with bursts of 3 APs (right). The bold line highlights one exemplary sensory-evoked response. The panels are those from **Fig. 5e**, but here scaled to match the scale of the *in vivo* data from panel a.

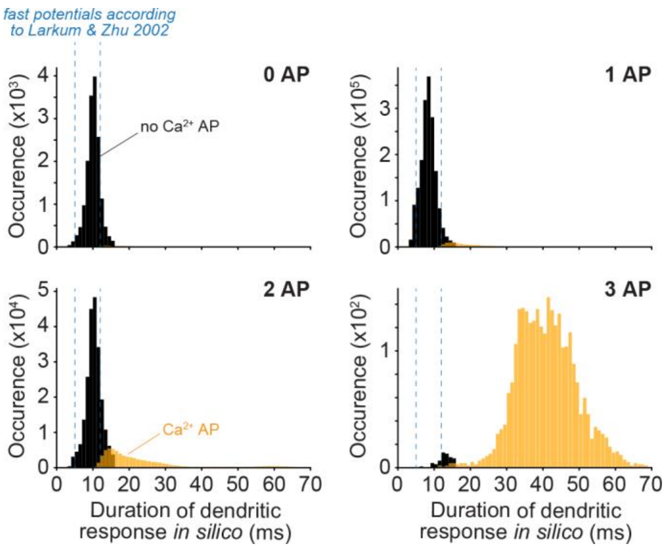

Fig. S7 (related to Fig. 5): *In silico* dendritic response versus somatic response. Histograms of the durations of sensory-evoked dendritic potentials for simulation trials without somatic responses (i.e., 0 AP), or with 1 AP responses, or bursts of 2 or 3 APs, respectively. Orange are trials that we identified as Ca^{2+} APs. Black are trials that we did not identify as Ca^{2+} APs. The blue dashed lines represent an *in vivo* definition of *fast* dendritic potentials[10] – i.e., dendritic potentials with durations of 12-18 ms were defined as *slow* and those longer than 18 ms as *complex* potentials[10]. Thus, a vast majority (>98%) of the sensory-evoked dendritic potentials that we predicted *in silico* to occur during somatic bursts of 3 APs (bottom right) resembled complex potentials (all of which we had identified as Ca^{2+} APs), some resembled slow potentials (most of which we had identified as no Ca^{2+} APs), and virtually none resembled fast potentials. In contrast, virtually none of the dendritic responses that we predicted for responses with 0 or 1 somatic APs resembled complex potentials, some resembled slow potentials, but the vast majority resembled fast potentials. Also for bursts of 2 APs, only a small minority of the dendritic responses resembled complex potentials. Thus, we predict that sensory-evoked bursts of 3 APs in PTs are a reliable readout for complex potentials in their apical dendrites – all of which we identified as Ca^{2+} APs generated via TC coupling (n = 2204552 simulation trials of the example morphology).

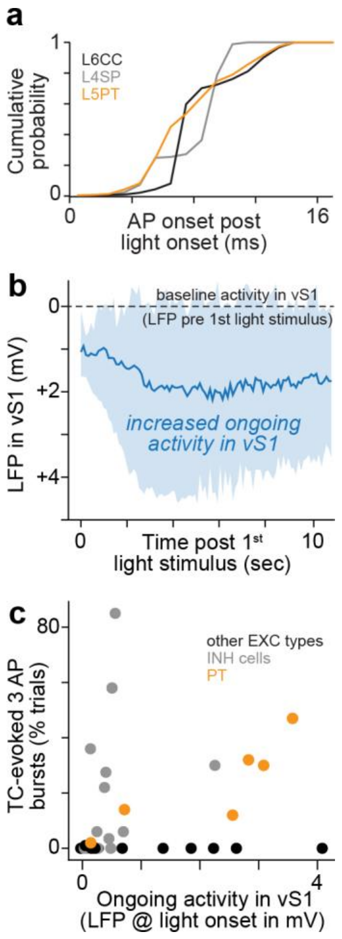

Fig. S8 (related to Fig. 10): Control data for optogenetic activation of TC synapses *in vivo*. a. Onsets of light-evoked APs in L6CCs, L4SPs and PTs at (n=6,721 trials, N=39 cells). **b.** Average LFP (solid blue line) and STD (light blue) in experiments in which we observed TC-driven bursts of 3 APs (n=750 trials, N=6 PTs). **c.** Light stimulated TC synapses evoke 3 AP bursts in both PTs and INH neurons, but not in neurons of other EXC types (here: L4SP, L5IT and L6CC). The fraction of trials in which PTs respond with 3 AP bursts increases with ongoing cortical activity. We did not observe such a relationship for light-evoked 3 AP bursts in INH neurons.

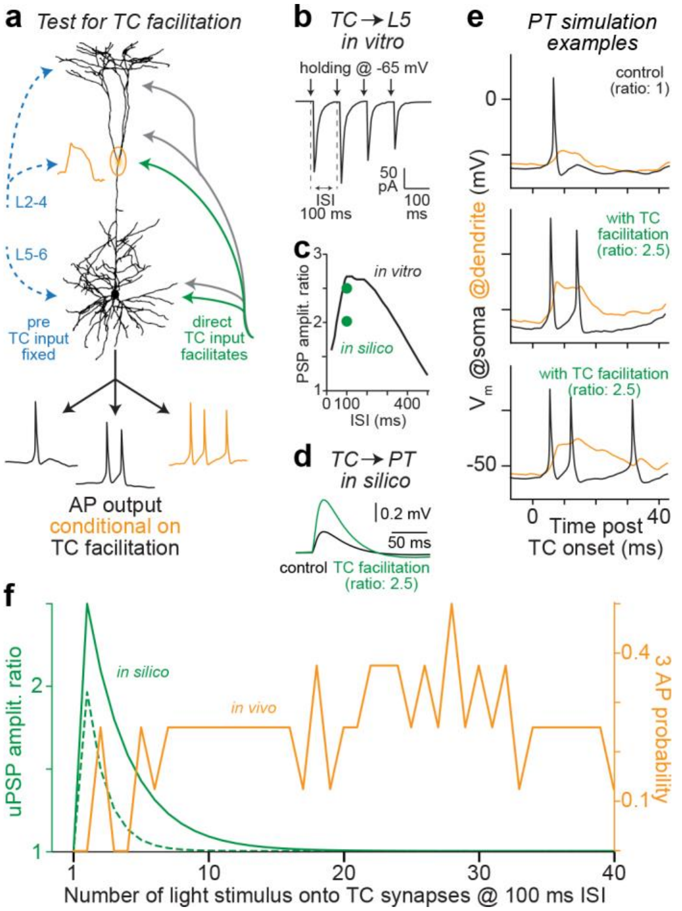

Fig. S9 (related to Fig. 9): Control data for optogenetic activation of TC synapses *in vivo*. a. We had stimulated TC synapses with light at inter-stimulus-intervals of 100 ms. Transitions from 1 APs into bursts of 2 or 3 APs that we had observed *in vivo* during our optogenetic experiments could hence reflect the facilitation of TC synapses[11]. We hence performed additional simulations to test this possibility as illustrated by this schematic. **b-d.** We incorporated the properties of facilitation, as observed *in vitro*[11], into the TC synapses of our PTs models. **e.** Simulation examples show how *in silico* manipulations of TC facilitation can affect responses with 1 AP (top) when we scaled uPSP of TC synapses by a factor of 2.5. Center panel shows example where TC facilitation led to transition into burst of 2 APs (without Ca^{2+} APs). Bottom panel shows example where TC facilitation led to transition into burst of 3 APs (with Ca^{2+} APs). **f.** Because TC synapses switch from facilitation to depression after a second stimulus (see panel b)[11], our simulations predicted (green) that bursts of 3 APs should increase the most for a second light stimulus and then decay rapidly within 10-15 light stimuli. Our *in vivo* recordings did not support this prediction (orange).

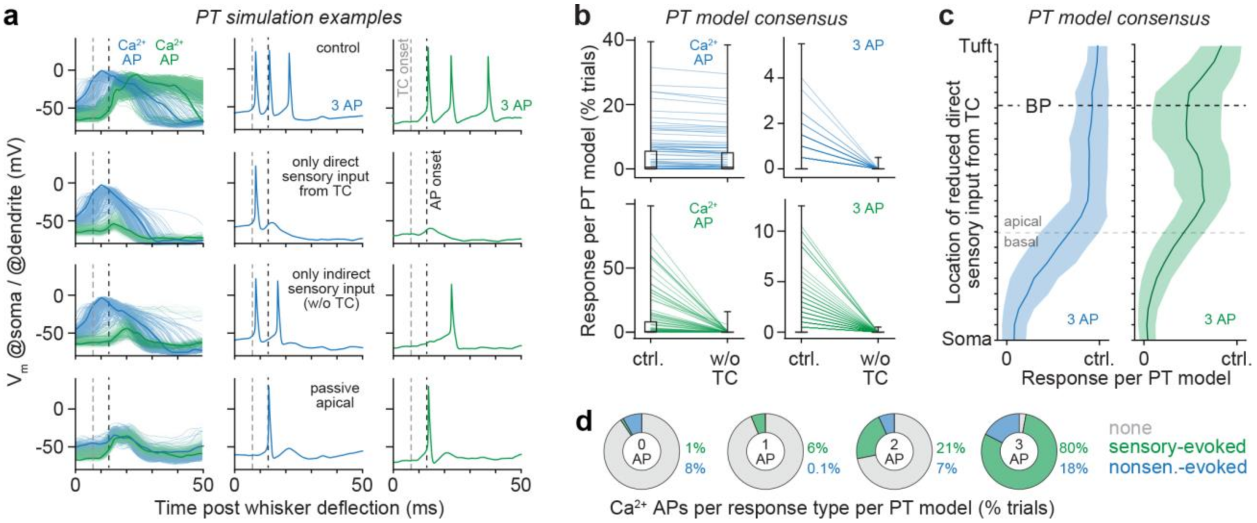

Fig. S10 (related to Fig. 9): *In silico* evidence for bursts in sensory-evoked cortical output via nonsensory-evoked Ca^{2+} APs. a. Simulation examples (n = 1000 trials) show two types of Ca^{2+} APs during bursts of 3 APs: Ca^{2+} APs that are abolished by removing direct or indirect sensory input (i.e., sensory-evoked Ca^{2+} APs, green) and those that persist (i.e., nonsensory-evoked Ca^{2+} APs, blue). Note that removing direct or indirect sensory input also abolishes bursts of 3 APs in trials with nonsensory-evoked Ca^{2+} APs. Thus, input from other long-range pathways to the apical dendrites can facilitate the generation of Ca^{2+} APs, and hence of bursts of 3 APs, but these bursts would have an early onset than those that originate via TC coupling. **b.** Model consensus: removing direct sensory input abolishes sensory-evoked Ca^{2+} APs, but not nonsensory-evoked Ca^{2+} APs, whereas removing direct sensory input abolishes bursts of 3 APs for both types of Ca^{2+} APs (i.e., sensory-evoked bursts of 3 APs are in general a reliable readout for Ca^{2+} APs) (n = 2,517,700 trials from all model configurations, which exhibit burst responses for the PT morphology shown in **Fig. 3**). **c.** Model consensus for configurations with 3 AP burst rates ≥2% for the PT morphology shown in **Fig. 3** (n=880): both 3 AP burst types require direct sensory input to basal dendrites, whereas bursts of 3 APs with sensory-evoked Ca^{2+} APs require also direct sensory input to the apical dendrites – in particular around the primary BP – i.e., they reflect TC coupling. Shadings represent the 25th to 75th percentiles, bold lines represent the medians. **d.** Input from other long-range pathways to the apical dendrites could facilitate the generation of nonsensory-evoked Ca^{2+} APs, which would result in 'early' bursts of 3 APs. We predict that nonsensory-evoked Ca^{2+} APs, and hence 'early' bursts occur very rarely during our experimental condition. Indeed, we did not observe such 'early' bursts *in vivo*. Thus, input from other long-range pathways to the apical dendrites could in principle facilitate bursts in sensory-evoked cortical output. However, TC coupling remains the mechanism that accounts for the vast majority of burst responses that we observed *in vivo* for both anesthetized and awake rats (n=1000 trials per somatic response, the trials are the same as in panel A).

Supplementary References

1. Paxinos G, Watson C. *The Rat Brain in Stereotaxic Coordinates*, Sixth Edition edn. Academic Press, Inc. (2006).
2. Rojas-Piloni G, Guest JM, Egger R, Johnson AS, Sakmann B, Oberlaender M. Relationships between structure, in vivo function and long-range axonal target of cortical pyramidal tract neurons. *Nat Commun* **8**, 870 (2017).
3. Oberlaender M, Ramirez A, Bruno RM. Sensory experience restructures thalamocortical axons during adulthood. *Neuron* **74**, 648-655 (2012).
4. Egger R, *et al.* Cortical Output Is Gated by Horizontally Projecting Neurons in the Deep Layers. *Neuron* **105**, 122-137 e128 (2020).
5. Oberlaender M, *et al.* Cell type-specific three-dimensional structure of thalamocortical circuits in a column of rat vibrissal cortex. *Cereb Cortex* **22**, 2375-2391 (2012).
6. Narayanan RT, *et al.* Beyond Columnar Organization: Cell Type- and Target Layer-Specific Principles of Horizontal Axon Projection Patterns in Rat Vibrissal Cortex. *Cereb Cortex* **25**, 4450-4468 (2015).
7. Hay E, Hill S, Schurmann F, Markram H, Segev I. Models of neocortical layer 5b pyramidal cells capturing a wide range of dendritic and perisomatic active properties. *PLoS Comput Biol* **7**, e1002107 (2011).
8. Constantinople CM, Bruno RM. Deep cortical layers are activated directly by thalamus. *Science* **340**, 1591-1594 (2013).
9. Helmchen F, Svoboda K, Denk W, Tank DW. In vivo dendritic calcium dynamics in deep-layer cortical pyramidal neurons. *Nat Neurosci* **2**, 989-996 (1999).
10. Larkum ME, Zhu JJ. Signaling of layer 1 and whisker-evoked Ca2+ and Na+ action potentials in distal and terminal dendrites of rat neocortical pyramidal neurons in vitro and in vivo. *J Neurosci* **22**, 6991-7005 (2002).
11. Viaene AN, Petrof I, Sherman SM. Synaptic properties of thalamic input to the subgranular layers of primary somatosensory and auditory cortices in the mouse. *J Neurosci* **31**, 12738-12747 (2011).
