# Supplementary material for: Thalamus enables active dendritic coupling of inputs arriving at different cortical layers
Source: Nat Commun. 2025 Sep 19;16:8327. doi: 10.1038/s41467-025-64152-0 (PMC12449479; doi:10.1038/s41467-025-64152-0)
Supplement: Supplementary file 2 — Description of Additional Supplementary Files [file 41467_2025_64152_MOESM2_ESM.pdf]

Description of Additional Supplementary Files

File name: Supplementary Movie 1
Description: Transformation of sensory input into cortical output *in silico*. The movie shows the simulated membrane potentials in response to passive whisker deflections across the dendrites of one example neuron model embedded into one example configuration of a network model of the rat barrel cortex (vS1). This multi-scale example model was generated for one of the layer 5 (L5) pyramidal tract neurons (PTs) in vS1 that we had first recorded and then labeled with Biocytin *in vivo*. In the first part of the movie, we show three example simulation trials that result in a whisker-evoked somatic response with a single action potential (AP), or with a burst of either 2 or 3 APs. For the network model, we show only the somata of those neurons that are presynaptic to the PT model and that elicited APs during the simulation period (i.e., active cells). For the PT model, we show only those synapses that correspond to the active cells in the network (i.e., active synapses). We colored active cells and synapses according to their cell types in thalamus and barrel cortex. In the second part of the movie, the synaptic input pattern resulting in a burst of 3 APs was replayed for different manipulations: (1) we removed the direct sensory input that the PT receives onto its dendrites from the whisker-related primary thalamus (VPM) – i.e., we deprived the PT model from all sensory-evoked thalamocortical (TC) synaptic inputs, (2) in addition to the direct sensory input, we also removed the indirect sensory input that the PT receives from corticocortical neurons at the L5-L6 border (i.e., L6CCs) – i.e., we deprived the PT model from all TC and L6CC synaptic inputs, (3) we removed all active conductances from the apical dendrite of the PT model (i.e., passive dendrites). We selected these three simulation trials as examples, because they were representative for the model consensus predictions from Figures 4, 5 and S5. The schematic of the rat at the beginning of the movie was adapted and modified from “Diamond ME, von Heimendahl M, Knutsen PM, Kleinfeld D, Ahissar E. 'Where' and 'what' in the whisker sensorimotor system. Nat Rev Neurosci 9, page 602, 2008, Springer Nature”. Reproduced with permission from Springer Nature.
