# Supplementary material for: Thalamus enables active dendritic coupling of inputs arriving at different cortical layers
Source: Nat Commun. 2025 Sep 19;16:8327. doi: 10.1038/s41467-025-64152-0 (PMC12449479; doi:10.1038/s41467-025-64152-0)
Supplement: Supplementary file 4 — Reporting Summary [file 41467_2025_64152_MOESM4_ESM.pdf]

nature portfolio

Corresponding author(s): Marcel Oberlaender

Last updated by author(s): Aug 6, 2025

# Reporting Summary

Nature Portfolio wishes to improve the reproducibility of the work that we publish. This form provides structure for consistency and transparency in reporting. For further information on Nature Portfolio policies, see our Editorial Policies and the Editorial Policy Checklist.

## Statistics

For all statistical analyses, confirm that the following items are present in the figure legend, table legend, main text, or Methods section.

| n/a | Confirmed | |
|---|---|---|
| ☐ | ☒ | The exact sample size (*n*) for each experimental group/condition, given as a discrete number and unit of measurement |
| ☐ | ☒ | A statement on whether measurements were taken from distinct samples or whether the same sample was measured repeatedly |
| ☐ | ☒ | The statistical test(s) used AND whether they are one- or two-sided<br>*Only common tests should be described solely by name; describe more complex techniques in the Methods section.* |
| ☒ | ☐ | A description of all covariates tested |
| ☐ | ☒ | A description of any assumptions or corrections, such as tests of normality and adjustment for multiple comparisons |
| ☐ | ☒ | A full description of the statistical parameters including central tendency (e.g. means) or other basic estimates (e.g. regression coefficient) AND variation (e.g. standard deviation) or associated estimates of uncertainty (e.g. confidence intervals) |
| ☐ | ☒ | For null hypothesis testing, the test statistic (e.g. *F*, *t*, *r*) with confidence intervals, effect sizes, degrees of freedom and *P* value noted<br>*Give P values as exact values whenever suitable.* |
| ☒ | ☐ | For Bayesian analysis, information on the choice of priors and Markov chain Monte Carlo settings |
| ☒ | ☐ | For hierarchical and complex designs, identification of the appropriate level for tests and full reporting of outcomes |
| ☐ | ☒ | Estimates of effect sizes (e.g. Cohen's *d*, Pearson's *r*), indicating how they were calculated |

*Our web collection on statistics for biologists contains articles on many of the points above.*

## Software and code

Policy information about availability of computer code

| | |
|---|---|
| Data collection | Spike2, LabView, Leica Matrix Screener, In Silico Framework |
| Data analysis | Amira, In Silico Framework |

For manuscripts utilizing custom algorithms or software that are central to the research but not yet described in published literature, software must be made available to editors and reviewers. We strongly encourage code deposition in a community repository (e.g. GitHub). See the Nature Portfolio guidelines for submitting code & software for further information.

## Data

Policy information about availability of data

All manuscripts must include a data availability statement. This statement should provide the following information, where applicable:

- Accession codes, unique identifiers, or web links for publicly available datasets
- A description of any restrictions on data availability
- For clinical datasets or third party data, please ensure that the statement adheres to our policy

Data Availability
All data are publicly available and source data are provided with this paper. The neuroanatomical and electrophysiological data were deposited in Edmond: https://doi.org/10.17617/3.ST7EWL. A minimal simulation dataset and code to reproduce the simulations in this study is available at https://github.com/mpinb/in_silico_framework_hot_zone. The full simulation dataset (>0.5 PB of data) can be made available upon request.

Code Availability
Al code and the custom-designed software to run simulations (In Silico Framework) are publicly available. The instructions for installing and running ISF, comprehensive documentations, tutorials and examples, all analyses routines, as well animated visualization of simulations and analyses are accessible via: https://wwwuser.gwdguser.de/~b.meulemeester/index.html. The code for generating, simulating and analyzing all multi-scale models is accessible for download via: https://github.com/mpinb/in_silico_framework_hot_zone. This repository comprises also a demo version of the code to reproduce the simulation and in silico manipulation examples in Fig. 4-6.

## Research involving human participants, their data, or biological material

Policy information about studies with human participants or human data. See also policy information about sex, gender (identity/presentation), and sexual orientation and race, ethnicity and racism.

| | |
|---|---|
| Reporting on sex and gender | *Use the terms sex (biological attribute) and gender (shaped by social and cultural circumstances) carefully in order to avoid confusing both terms. Indicate if findings apply to only one sex or gender; describe whether sex and gender were considered in study design; whether sex and/or gender was determined based on self-reporting or assigned and methods used. Provide in the source data disaggregated sex and gender data, where this information has been collected, and if consent has been obtained for sharing of individual-level data; provide overall numbers in this Reporting Summary. Please state if this information has not been collected. Report sex- and gender-based analyses where performed, justify reasons for lack of sex- and gender-based analysis.* |
| Reporting on race, ethnicity, or other socially relevant groupings | *Please specify the socially constructed or socially relevant categorization variable(s) used in your manuscript and explain why they were used. Please note that such variables should not be used as proxies for other socially constructed/relevant variables (for example, race or ethnicity should not be used as a proxy for socioeconomic status). Provide clear definitions of the relevant terms used, how they were provided (by the participants/respondents, the researchers, or third parties), and the method(s) used to classify people into the different categories (e.g. self-report, census or administrative data, social media data, etc.) Please provide details about how you controlled for confounding variables in your analyses.* |
| Population characteristics | *Describe the covariate-relevant population characteristics of the human research participants (e.g. age, genotypic information, past and current diagnosis and treatment categories). If you filled out the behavioural & social sciences study design questions and have nothing to add here, write "See above."* |
| Recruitment | *Describe how participants were recruited. Outline any potential self-selection bias or other biases that may be present and how these are likely to impact results.* |
| Ethics oversight | *Identify the organization(s) that approved the study protocol.* |

Note that full information on the approval of the study protocol must also be provided in the manuscript.

# Field-specific reporting

Please select the one below that is the best fit for your research. If you are not sure, read the appropriate sections before making your selection.

☒ Life sciences ☐ Behavioural & social sciences ☐ Ecological, evolutionary & environmental sciences

For a reference copy of the document with all sections, see nature.com/documents/nr-reporting-summary-flat.pdf

# Life sciences study design

All studies must disclose on these points even when the disclosure is negative.

| | |
|---|---|
| Sample size | no sample-size calculation was performed |
| Data exclusions | no data was excluded form the analysis |
| Replication | all reconstructions of synapse distributions on 10 PT neurons yielded the same result |
| Randomization | no experimental groups, thus does not apply |
| Blinding | no experimental groups, thus does not apply |

# Reporting for specific materials, systems and methods

We require information from authors about some types of materials, experimental systems and methods used in many studies. Here, indicate whether each material, system or method listed is relevant to your study. If you are not sure if a list item applies to your research, read the appropriate section before selecting a response.

## Materials & experimental systems

| n/a | Involved in the study |
| --- | --- |
| ☐ | ☒ Antibodies |
| ☒ | ☐ Eukaryotic cell lines |
| ☒ | ☐ Palaeontology and archaeology |
| ☐ | ☒ Animals and other organisms |
| ☒ | ☐ Clinical data |
| ☒ | ☐ Dual use research of concern |
| ☒ | ☐ Plants |

## Methods

| n/a | Involved in the study |
| --- | --- |
| ☒ | ☐ ChIP-seq |
| ☒ | ☐ Flow cytometry |
| ☒ | ☐ MRI-based neuroimaging |

# Antibodies

Antibodies used

1) rabbit anti-mCherry (Invitrogen #PA5-34974 & M11217); 2) mouse anti-VGlut2 (Synaptic Systems #135421); 3) goat anti-Rabbit IgG Alexa-647 H+L (Invitrogen #A21245); 4)goat anti-Mouse IgG Alexa-405 H+L (Invitrogen #A31553).

Validation

1) rabbit anti_mCherry (Invitrogen #PA5-34974 & M11217): validation from manufacturer's website: "Immunofluorescent analysis of mCherry Tag was performed using 70% confluent construct name transfected HEK-293 cells. The cells were fixed with 4% paraformaldehyde for 10 minutes, permeabilized with 0.1% Triton™ X-100 for 15 minutes, and blocked with 2% BSA for 1 hour at room temperature. The cells were labeled with mCherry Polyclonal Antibody (Product # PA5-34974) at 1:100 dilution and Histone H3 Monoclonal Antibody (865R2) (Product # AHO1432) at 1:200 dilution in 0.1% BSA, incubated at 4°C overnight and then labeled with Goat anti-Rabbit IgG (H+L) Highly Cross-Adsorbed Secondary Antibody, Alexa Fluor Plus 647 (Product # A32733) and Goat anti-Mouse IgM (Heavy chain) Cross-Adsorbed Secondary Antibody, Alexa Fluor 488 (Product # A-21042) respectively at a dilution of 1:2,000 for 45 minutes at room temperature. Panel a (Nuclei: Red) represents mCherry Tag. Panel b (Nuclei: Green) represents Histone H3. Panel c (Nuclei: Blue) represents ProLong™ Diamond Antifade Mountant with DAPI (Product # P36962). Panel d represents the merged image showing the co-localization of nuclear signals in transfected cells. Panel e represents untransfected HEK-293 cells. Panel f represents control cells with no primary antibody to assess background. The images were captured at 60X magnification.
References for validation: Dias et al J Neuroscience 2021.; Rogers-Carter et al J Neuroscience 2019.

2) mouse anti-VGlut2 (Synaptic Systems #135421):
references for validation: Fremeau RT et al Neuron 2001; Takamori S et al J Neuroscience 2001

3) goat anti-Rabbit Alexa-647 (Invitrogen #A21245) validation from manufacturer's website "To minimize cross-reactivity, these goat anti-rabbit IgG whole antibodies have been cross-adsorbed against bovine IgG, goat IgG, mouse IgG, rat IgG, and human IgG. Cross-adsorption or pre-adsorption is a purification step to increase specificity of the antibody resulting in higher sensitivity and less background staining. The secondary antibody solution is passed through a column matrix containing immobilized serum proteins from potentially cross-reactive species. Only the nonspecific-binding secondary antibodies are captured in the column, and the highly specific secondaries flow through. The benefits of this extra step are apparent in multiplexing/multicolor-staining experiments (e.g., flow cytometry) where there is potential cross-reactivity with other primary antibodies or in tissue/cell fluorescent staining experiments where there are may be the presence of endogenous immunoglobulins. Anti-Rabbit secondary antibodies are affinity-purified antibodies with well-characterized specificity for rabbit immunoglobulins and are useful in the detection, sorting or purification of its specified target. Secondary antibodies offer increased versatility enabling users to use many detection systems (e.g. HRP, AP, fluorescence). They can also provide greater sensitivity through signal amplification as multiple secondary antibodies can bind to a single primary antibody. Most commonly, secondary antibodies are generated by immunizing the host animal with a pooled population of immunoglobulins from the target species and can be further purified and modified (i.e. immunoaffinity chromatography, antibody fragmentation, label conjugation, etc.) to generate highly specific reagents.

references for validation: Ashraf, HM., fernandez, B., et al Nature Communications 2023; York, HM., Joshi,K., et al Nature Communications 2023.

4) goat anti-Mouse IgG Alexa-405 H+L (Invitrogen #A31553) validation from manufacturer's website " Anti-Mouse secondary antibodies are affinity-purified antibodies with well-characterized specificity for mouse immunoglobulins and are useful in the detection, sorting or purification of its specified target. Secondary antibodies offer increased versatility enabling users to use many detection systems (e.g. HRP, AP, fluorescence). They can also provide greater sensitivity through signal amplification as multiple secondary antibodies can bind to a single primary antibody. Most commonly, secondary antibodies are generated by immunizing the host animal with a pooled population of immunoglobulins from the target species and can be further purified and modified (i.e. immunoaffinity chromatography, antibody fragmentation, label conjugation, etc.) to generate highly specific reagents."

references for validation: Sadhanasatish, T et al Science Advances 2023; Trotter JH et al Nature Communications 2023.

# Animals and other research organisms

Policy information about studies involving animals; ARRIVE guidelines recommended for reporting animal research, and Sex and Gender in Research

Laboratory animals

Wistar rats (P22-P70, male)

Wild animals

the study did not involve wild animals

| | |
|---|---|
| Reporting on sex | All animals included in the study were male |
| Field-collected samples | the study did not contain samples collected from the field |
| Ethics oversight | All experiments were carried out after evaluation by the local German or Dutch authorities, and in accordance with the animal welfare guidelines of the Max Planck Society. |

Note that full information on the approval of the study protocol must also be provided in the manuscript.

## Plants

| | |
|---|---|
| Seed stocks | *Report on the source of all seed stocks or other plant material used. If applicable, state the seed stock centre and catalogue number. If plant specimens were collected from the field, describe the collection location, date and sampling procedures.* |
| Novel plant genotypes | *Describe the methods by which all novel plant genotypes were produced. This includes those generated by transgenic approaches, gene editing, chemical/radiation-based mutagenesis and hybridization. For transgenic lines, describe the transformation method, the number of independent lines analyzed and the generation upon which experiments were performed. For gene-edited lines, describe the editor used, the endogenous sequence targeted for editing, the targeting guide RNA sequence (if applicable) and how the editor was applied.* |
| Authentication | *Describe any authentication procedures for each seed stock used or novel genotype generated. Describe any experiments used to assess the effect of a mutation and, where applicable, how potential secondary effects (e.g. second site T-DNA insertions, mosiacism, off-target gene editing) were examined.* |
